# An overview of the toxic effect of potential human carcinogen Microcystin-LR on testis

**DOI:** 10.1016/j.toxrep.2015.01.008

**Published:** 2015-01-27

**Authors:** Yaqoob Lone, Raj Kumar Koiri, Mangla Bhide

**Affiliations:** Department of Zoology, Dr. Harisingh Gour Central University, Sagar, Madhya Pradesh 470003, India

**Keywords:** Microcystins, Microcystin-LR (MC-LR), Testis, DNA damage, Oxidative stress, Spermatogenesis

## Abstract

The worldwide occurrence of cyanobacterial blooms due to water eutrophication evokes extreme concerns. These blooms produce cyanotoxins which are hazardous to living organisms. So far among these toxins, Microcystin-LR (MC-LR) is the most toxic and the most frequently encountered toxin produced by the cyanobacteria in the contaminated aquatic environment. Microcystin-LR is a potential carcinogen for animals and humans, and the International Agency for Research on Cancer has classified Microcystin-LR as a possible human carcinogen. After liver, testis has been considered as one of the most important target organs of Microcystin-LR toxicity. Microcystin-LR crosses the blood–testis barrier and interferes with DNA damage repair pathway and also increases expression of the proto-oncogenes, genes involved in the response to DNA damage, cell cycle arrest, and apoptosis in testis. Toxicity of MC-LR disrupts the motility and morphology of sperm and also affects the hormone levels of male reproductive system. MC-LR treated mice exhibit oxidative stress in testis through the alteration of antioxidant enzyme activity and also affect the histopathology of male reproductive system. In the present review, an attempt has been made to comprehensively address the impact of MC-LR toxicity on testis.

## Graphical abstract

**Figure:**
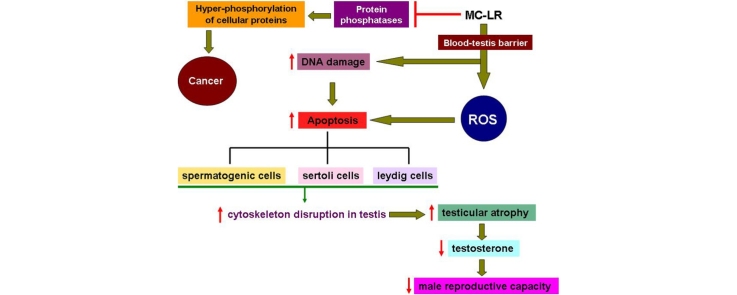

## 1 Introduction

**Fig. 1:**
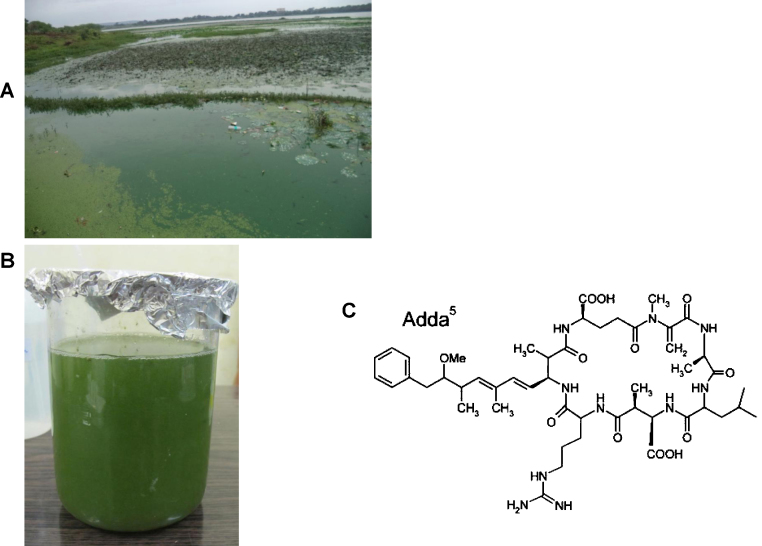
*Microcystis aeruginosa* bloom in Sagar lake water (A), sample of water containing *Microcystis aeruginosa* from a contaminated water reservoir (B), structure of microcystin-LR (C).

Cyanobacteria are found in fresh, brackish and marine water bodies throughout the world. Many species of these bacteria are capable of producing toxins (cyanotoxins), most of which are released after cell death. Microcystins, specific hepatotoxins produced by several cyanobacteria species in eutrophic surface waters, have received increasing worldwide concern in the past decade because of their toxic potential. The microcystins are monocyclic heptapeptides composed of d-alanine at position 1, two variable l-amino acids at positions 2 and 4, g-linked d-glutamic acid at position 6, and 3 unusual amino acids: b-linked d-erythro-b-methylaspartic acid (MeAsp) at position 3;(2S,3S,8S,9S)-3-amino-9-methoxy-2,6,8-trimethyl-10-phenyldeca-4,6-dienoic acid (Adda) at position 5; and N-methyl dehydroalanine (MDha) at position 7. The unusual amino acid Adda is essential for expression of biological activity, and a different stereochemistry about the conjugated double bond, for example, results in abolition of toxicity [1], [2], [3]. More than 90 microcystin isoforms have been detected, among which microcystin-leucine arginine (MC-LR) is the most abundant (Fig. 1) and the most toxic variant of microcystin [4].

**Fig. 2:**
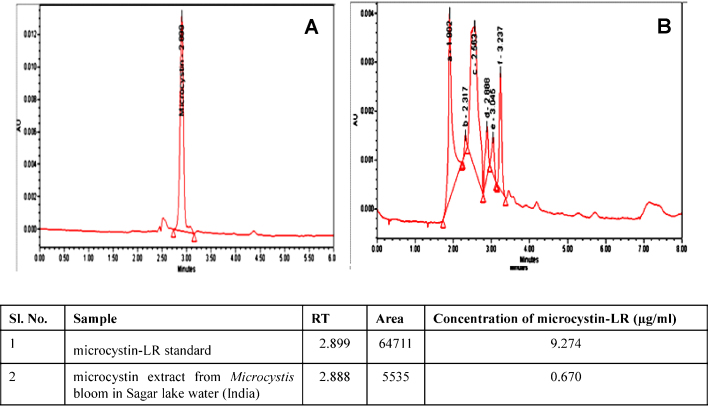
HPLC chromatograms of (A) microcystin-LR standard; (B) microcystin extract containing microcystin-LR from microcystis bloom in Sagar lake water and were determined at PDA 238 nm. The injection volume was 20 μl and the retention time of Microcystin-LR was around 2.9.

For many years, the existence of cyanobacterial blooms, especially microcystins in drinking water, have resulted in a number of public health events [5]. It is well known that microcystins can bioaccumulate in aquatic animals ([6], [7], [8]) and these toxins can be transferred along the food web to high trophic levels, even to human beings [6]; hence consumption of aquatic animals containing MCs represents potential risk to human health. As the source of drinking water, more and more water bodies are facing the problem of MC-LR pollution [9], [10]. The problem due to MC-LR gets compounded by the fact that it is concentrated by boiling, thus increasing risk and it is also resistant to chemical hydrolysis or oxidation at near-neutral pH ([11], [12], [13]). Moreover, Wannemacher [14] reported that MC-LR is stable even at temperatures up to 300 °C in laboratory conditions. The provisional guideline set by the World Health Organization (WHO) for Microcystin-LR (MC-LR) in drinking water is 1 μg/L, but the concentration of MCs in many water bodies is far beyond that guideline, *e.g.*, in Sagar lake water (India) Microcystin-LR was found to be 0.67 μg/ml (Fig. 2). Microcystin-LR is a potential carcinogen for animals and humans, and the International Agency for Research on Cancer has classified Microcystin-LR as a possible human carcinogen due to its potential carcinogenic activity *via* inhibition of protein phosphatases, which leads to the hyper-phosphorylation of cellular proteins [15].

## 2 MC-LR induced toxicity on rodents spermatogenesis

Liver is the most important target organ of microcystin. It also affects the heart, kidney, nervous system, gastrointestinal tract and exhibits genotoxicity [16]. A few studies have indicated that microcystin are accumulated in the gonads of invertebrates and thus, gonads are considered as second target organ of microcystin [17]. A few studies have shown that MCs were toxic to the male reproductive system and in particular the testes were more sensitive than the liver or other organs [18]. Studies have confirmed that MCs induce germ cell apoptosis associated with the mitochondrial-dependent apoptotic pathway [19]. Li et al. [18] stated that male rats exposed to MC-LR doses of 5, 10 or 15 μg/kg exhibited or resulted in decreased testicular weight and epididymal sperm concentration.

The hypothalamic–pituitary–gonadal axis is an instrumental pathway for endocrine regulation and proper function, where hypothalamic gonadotrophin releasing hormone (GnRH) stimulates the release of follicle-stimulating hormone (FSH) and luteinizing hormone (LH) from the pituitary and plays a key role in the neuro-hormonal control of reproduction [20]. MC-LR has been reported to modulate the hypothalamic–pituitary–gonadal axis *via* damaging and causing apoptosis of the Leydig cells of testis, impairment of spermatogenesis and causing changes in serum testosterone level and other hormones [18], [21].

Male rats were treated with MC-LR (i.p.) at a dose of 0, 5, 10 or 15 μg/kg b.w. for 28 days. The study showed that exposure to 5 μg/kg b.w. of MC-LR decreased the sperm motility and increased the sperm abnormality rate, and exposure to 15 μg/kg b.w. of MC-LR decreased testis weight, sperm concentration, the levels of serum testosterone, FSH and LH. The histological detection showed that the seminiferous tubules atrophied and obstructed [18]. Chen et al. [22] observed that after 50 days exposure of 10 μg/kg b.w. of MC-LR in rats, a significant decrease in testes index was observed in high dose group and this decline was also consistent with the results of testicular atrophy in morphological observation. Thus, MC-LR had prominent toxic effects on male reproductive capacity, and the toxic effects were evident by prolongation of exposure time. MC-LR could cause apoptosis of primary cultured spermatogenic cells, Sertoli cells, and Leydig cells, with Leydig cells being the most sensitive to MC-LR toxicity [18].

Male mice were exposed orally to chronic low-dose of microcystins at 0, 1, 3.2 and 10 μg/L for 3 and 6 months. The study showed that sperm quality declined at 3.2 and 10 μg/L, testosterone levels decreased at 10 μg/L, LH and FSH levels increased, and apoptosis of Leydig cells occurred in three-month group. The changes of sperm abnormality rate and testosterone level in six-month group were similar to the three-month group, but these changes were more marked. It was noted that the testis structural impairment was observed at 10 μg/L dose in six month group. Therefore, these results implied that chronic low-dose MC-LR treatment can lead to toxicity to testis and affect the hormone level [23]. Thus, it is evident that animals and humans routinely ingest food or liquids contaminated with MC-LR, which in turn is expected to enter and accumulate in testis and exert toxicity to male reproductive system [23]. Male mice treated with microcystin intraperitoneally to 3.33 or 6.67 mg/kg body weight for 14 days showed that sperm viability and sperm motility (specifically, rapid progressive motility) decreased significantly compared to the control. Results also demonstrated that the absolute weights of both testes and epididymides from male mice treated with 6.67 mg microcystins/kg/day dose were significantly decreased in comparison to the control, indicating that microcystins have a toxic impact on testes and epididymides [24].

## 3 MC-LR effect the histopathology of the male reproductive system

Intraperitoneal administration of an acute dose of MC-LR (300 μg/kg b.w. for 6 days) has indicated that MC-LR enters spermatogonia and Sertoli cells but not Leydig cells, suggesting that reproductive toxicity of MC-LR were induced by its distribution in testis [25]. Apoptotic cells have been observed to be mainly present in the outermost portion or in the inner side of the seminiferous tube, where Sertoli cells and spermatogonic cells are present [25]. Ultrastructural observations of prepubertal rabbit testes exposed to MC-LR have been reported to show widened intercellular junctions and distention of the mitochondria, endoplasmic reticulum, and Golgi apparatus [26]. MC-LR can exert generally a chronic toxicity to male rat reproductive system through influencing the cytoskeleton and mitochondria on being treated with 1 or 10 μg/kg b.w. MC-LR for 50 days [22]. Treatment of mice with MC-LR at a dose of 5–10 μg/kg b.w. causes slight testicular atrophy associated with changes and blockage in seminiferous tubules, slight deformation of androgonial and spermatogenic cells, enlargement of the lumen of the seminiferous tubules, thinning of the spermatogenic epithelium as well as depopulation of Leydig cells, reduced numbers of interstitial cells, Sertoli cells and mature sperm (Table 1) [18], [23], [24], [25].

**Table 1:** Summary of MC-LR doses used and effects observed from both *in vitro* and *in vivo* studies.

Test organism/system	Method	Time of exposure	MC-LR concentration	Outcome	References
*In vitro studies*					
MC-LR distribution					
Primary cultured spermatogonia	Western blot LC-MS	6 h	500 nM	Presence of MC-LR	[25]
Sertoli cells		48 h		Presence of MC-LR	
Leydig cells		2 h		Not detected	

Apoptosis					
Sertoli cells	RT-PCR	24 h	0, *1 and *10 μg/ml	*Condensed chromatin and fragmented DNA	[32]
	Western blot				

Cytotoxicity					
Spermatogonia	Cell viability assay	6 h	0, 0.5*, 5*, 50* and 500* nmol/L	*Decreased significantly	[28]
	FDA and PI staining			*Apoptosis increased significantly	
	Antioxidant capacity			*Significantly decreased	
	ROS formation			*Increased significantly	
	Western blot			*Oatp 3a1 intensity increased	

Cytotoxicity					
Leydig cells	FDA and PI staining	12, *24^b^ and *48 h	0, 0.5_c_, *5 ^a^, *50 or *500^b^ nM	Decreased significantly *Apoptosis induced^a^ * ROS and LPO increases, ^a^*SOD decreases *Drops significantly^b^_c_	[18]
	Oxidative stress				
	Hormone estimation				

Cytotoxicity					
Primary rat sertoli cells	Cell viability assay^a^	6, 12, and 24 h^a^	0 μg/L, *0.15 μg/L, *1.5 μg/L and *15^c^ μg/L	*No significant difference	[38]
	LDH			*Increases slightly^c^	
	SOD			*Differs significantly^a^	
	ROS			*Increases significantly^c^	
	LPO			*No significant difference	

*In vivo studies*					
Mice testes and epididymides	Serum hormone assay	3 and *6 m	0, 1, *3.2 and *10^a^ μg/L (Orally)	*Testosterone level decreases	[23]
	Sperm analysis			*Sperm motility and count decreases	
	TUNEL staining			*Apoptosis occurs	
	Histopathological evaluation			*Testicular atrophy Lumen of the seminiferous tubules enlarges^a^	

Male mice testes	Micronucleus assay	14 days	0, 3,*6 and*12 μg/kg bw (peritoneal)	*Micronucleus rate increases	[36]
	DPC coefficient	7 days		*DPC coefficient increases	

Male rat testes	TUNEL staining	1, 2, 4.*6,*12 and *24^a^ h	*80.5 μg/kg bw (intravenous)	*Apoptosis increases significantly *Elevation of FasL and Fas, downstream effectors-FADD, caspase-8, Apaf-1, caspase-9 and caspase-3^a^	[34]
	Western blot				
	RT-PCR				

Male mice testes	Serum hormone assay	1, 4, 7 and 14 days	3.75, 7.5, 15 and 30 μg/kg bw (intraperitoneal)	Levels of FSH, testosterone, LH fluctuates with dose and duration FSHβ and LHβ expression varies with dose and duration GnRH was down regulated	[20]
	RT-PCR				

Male mice testes		13 h and 4 days 13 h	3.75, 7.5^a^, *15 and *30 μg/kg bw (intraperitoneal)	*Phosphorylation p53 and Bcl-2^a^ *Modulation of c-myc, c-jun, c-fos, Bax, caspase 3 and caspase 8 *Apoptosis occurs *Loss and derangement of spermatogonic cells Lumen enlargement, thinning of spermatogenic epithelium were observed	[25]
	RT PCR Western blot				
	TUNEL staining				
	Histopathological evaluation				

Male rat testes	TEM	50 days	1^a^ and *10 μg/kg bw (intraperitoneal)	*Condensation and margination of chromatin *Shrunk spermatogonia, mitochondria swollen	[22]
	Hormone assay			*FSH and LH increases significantly *Testosterone decreases	
	ROS			*ROS increases significantly	
	RT-PCR			*All 8 mitochondrial genes were elevated	

Male rat testes	Serum hormone assay	28 days	0,*5, *10 or *15^b^ μg/kg bw (intraperitoneal)	*FSH and LH increases and decreases in higher doses^b^ ROS and LPO increases *Enlargement of seminiferous tubules *Decreases significantly	[18]
	Sperm analysis				
	ROS and LPO				
	Histopathology				
	Testosterone level				

^*,a,b^ refers to the result obtained for the corresponding doses of MC-LR concentration used both *in vitro* and *in vivo* studies.

## 4 MC-LR transportation in testis

Organic anion transporting polypeptide superfamily (Oatps) has been reported to transport MCs into cells [27] and at least five kind of Oatp subunits (Oatp1a5, -3a1, -6b1, -6c1 and -6d1) [28] have been observed at the mRNA level in spermatogonia, and the expressions of these Oatps was influenced by MC-LR, especially the Oatp3a1 [28], [29] but they did not look at other Oatp predominantly expressed in testis like Oatp6a1 [30]. However, MC-LR uptake by Oatp into testis itself has not been demonstrated and it is premature to say that this is how (or the only way) MCs get into testis; still a lot is unknown about Oatps, tissue uptake, *etc.* Using immunofluorescence detection, it was observed that MC-LR passes into testis, when rats were injected intra-peritoneally with 300 μg/kg b.w. for 6 days and spread out on the tubal wall of seminiferous tubules, in which spermatogonia and Sertoli cells are mainly located [31]. The fact that MC-LR can enter testis was further confirmed when western blot analysis revealed MC-LR-protein phosphatase 1/2A (PP1/2A) adducts in the extracts from testes [31]. MC-LR modulates intracellular biochemical reactions by covalently binding with protein phosphatases1 and 2A (PP1/2A), eventually resulting in apoptosis in testes [18], [23]. Earlier Zhang et al. [32] has observed that the ultrastructure of testis shows some typical apoptotic features, including cell membrane blebbing, cytoplasmic shrinkage, swollen mitochondria, and deformation of the nucleus, when the rats were treated with 10 μg/kg b.w. and thus they concluded that MC-LR can pass through the blood–testis barrier (BTB) and cause morphological damage of testes.

## 5 MC-LR effects the expression of proto-oncogene; tumor suppressor and DNA damage response genes of male reproductive system

Apoptosis is an active process of cellular self destruction that requires the expression of specific genes including bax, bcl-2, p53 and caspase 3 [33]. The level of expression of p53 and pro apoptotic protein bax increases while the expression of anti-apoptotic protein bcl-2 decreases when Sertoli cells of rat were exposed with MC-LR suggesting that MC-LR induced apoptosis in Sertoli cells *via* modulating the expression of p53 and bcl-2 family proteins [32]. Similarly, rats exposed to MC-LR equivalent (from cyanobacterial crude extract) at a dose of 80.5 μg/kg b.w. for 1, 2, 4, 6, 12 and 24 h induced germ cell apoptosis in the testes due to up regulation of the mRNA expressions of Fas, FasL in 1, 2, 4 and 6 h exposure and the expression of their downstream effectors-FADD, caspase-8, Apaf-1, caspase-9 and caspase-3 changes within 24 h post-injection of MCs [34]. Another study has reported that when rats are exposed to 86.7 μg MC-LR/kg b.w. for 2, 4, 6, 12, 24 h, it results in the induction of the transcript of oncogene c-fos, c-jun and c-myc gene in kidney and testis. It was observed that there was also potential tumor-promoting activity in kidney and testis when exposed to MCs, although such potential was weaker in kidney and testis than in liver of rat [35]. Significant increases in the phosphorylation of both p53 and Bcl-2 were identified in testes after the administration of MC-LR at 7.5, 15 or 30 μg kg/b.w. for 4 times [25].

Cytoskeleton disruption is one of the hallmarks of cytotoxicity. MC-LR toxicity has been reported to cause cytoskeleton disruption in testis which in turn weakens the testosterone synthesis ability in rats [22] due to remarkable disruption of transcriptional balance/altered expression of some cytoskeletal genes like MFs, MTs and IFs, causing morphological changes, and toxicity to the reproductive system [22]. MC-LR has been reported to induce excessive DNA-protein crosslinks and increase the micronucleus rate significantly in the mice testicular cells as a function of MC-LR concentration [36].

## 6 MC-LR induces oxidative stress in testis

Numerous hypotheses have been proposed for MC-LR toxicity; however, in recent times, toxicity due to oxidative stress is attracting more attention. Reactive oxygen species (ROS) are chemically reactive molecules containing oxygen. Under normal conditions ROS plays an important role in the normal physiological functions of the reproductive system; however, when its level increases dramatically, it causes significant damage to cellular structures [37]. In order to explore the toxic effects of MC-LR on spermatogonia *in vitro*, spermatogonia were treated with 0, 0.5, 5, 50, and 500 nmol/L MC-LR for 6 h, resulted in decline of cell viability and total antioxidant capacity, whereas the ratio of apoptotic cells, reactive oxidative species (ROS) generation, mitochondrial membrane potential (MMP), and intracellular free Ca^(2+)^ increases leading to apoptosis [28]. Similarly exposure of Sertoli cells with 0.15, 1.5, 15 μg/L of MC-LR has been reported to cause higher concentration of ROS [38].

Superoxide dismutases are enzymes that catalyze the dismutation of superoxide (O_2_^−^) into oxygen and hydrogen peroxide. Experiments with Sertoli cells have shown that with increased concentration of MC-LR, level of SOD was reduced [38]. Thus, oxidative stress has been suggested to cause serious damage to testicular function [18]. Testis mtDNA is another target for MC-LR induced oxidative damage and if not repaired can lead to mitochondrial dysregulation and cell death [39] due to excessive ROS formation and transcriptional activation of mitochondrial genes [38].

Lipid peroxidation, which refers to the oxidative degradation of lipids, is one of the most common biomarkers used to indicate oxidative stress levels in animals [40]. Mitochondrial membranes contain large number of poly unsaturated fatty acids (PUFAS) in their phospholipids and are prime sites of lipid peroxidation [41]. The enhanced lipid peroxidation in testis may result in the disintegration of the mitochondrial membrane ultra structure which in turn affects the membrane bound LDH function [42]. It has been revealed that LDH in testicular tissue are associated with the maturation of germinal epithelial layer of seminiferous tubules [43]. The considerable decreased level of LDH suggests that the toxic exposure of MC-LR could cause deterioration of germinal epithelium and damage the testis.

Significant increase in the basal lipid peroxidation level as well as a decrease in the GSH/GSSG ratio has been considered as essential indicators of oxidative stress in cell compartments [44], [45]. Glutathione S-transferases (GST) are enzymes that are involved in the detoxification and elimination of peroxides that are formed during metabolism. GST involvement in the *in vivo* MC-LR detoxication pathways has been shown in several organisms [46] and its activity has been reported to be lower in testis of mice treated with 34.5 mg/kg b.w. of MC-LR [42].

## 7 MC-LR affects the male reproductive system of fishes and amphibians

Cyanobacterial cells release microcystins into the water bodies where aquatic organisms especially fish spend their whole life including growth, reproduction and embryonic development [47]. Liver is considered as the first target organ of MCs [48] but it can be transported through the blood to different organs such as gonads [48], [49] and it is noteworthy that MCs exert great harm to the reproductive system of fish subjected to cyanobacterial blooms [50]. After intraperitoneal injection with MC-LR, zebrafish testes showed widened intercellular spaces or junctions and distention of mitochondria [51]. MCs reduced fertility rate and survival of southern catfish *(Silurus meridionalis)* [47]. Fishes treated with 5 and 20 μg/L MC-LR for 30 days have shown cellular deterioration and optically empty intercellular spaces indicating testis lesions caused by reduced proportion of mature sperm. It also induced apoptosis through the mitochondrial pathway in the reproductive system of fish by down-regulation of Bcl-2 (anti-apoptotic gene) expression [52].

The attenuation of global amphibian populations has become a serious problem worldwide. The decline in the number of amphibian species has been widely reported [53], [54] and it has a major impact on other biological organisms because amphibians are an important part of the ecosystem [55]. *In vitro* studies have shown that spermatogenic Sertoli cells of *Rana nigromaculata* treated with 1 μg/L MC-LR shows typical ultrastructural changes such as swelling of the mitochondria and endoplasmic reticulum which are associated with necrosis [56]. Treatment of male frog testes with MC-LR also caused damage to spermatogenic Sertoli cells, thereby inducing reproductive toxicity [56]. It was observed that Bax was up regulated and Bcl-2 was down regulated following prolonged exposure to 1 μg/L MC-LR to *Rana nigromaculata*. The ratio of Bax to Bcl-2 also significantly increased in a time-dependent manner, thus contributing to MC-induced apoptotic cell death in frog testes [56]..

## 8 Conclusion

**Fig. 3:**
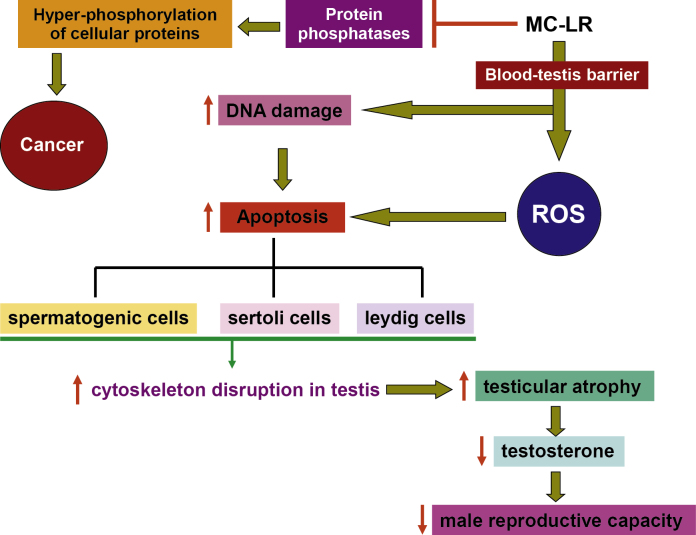
In testes, Microcystin-LR (MC-LR) crosses the blood–testis barrier and induces mitochondrial dependent apoptotic pathway in response to DNA damage and/or oxidative stress in spermatogenic cells, Sertoli cells and Leydig cells, resulting in disruption of cytoskeleton and testicular atrophy. At the hormonal level this results in decrease of testosterone level and overall decline in male reproductive potential. MC-LR also acts as a possible human carcinogen due to its potential carcinogenic activity *via* inhibition of protein phosphatases, which leads to the hyper-phosphorylation of cellular proteins.

Microcystin-LR is a cyclic heptapeptide which possesses the ability to inhibit the serine/threonine protein phosphatases PP1 and PP2A and, consequently, exhibits acute hepatocytotoxicity. However, little is known about the toxic effects of MC-LR on organs other than liver. In the present review, an attempt has been made to comprehensively address the impact of MC-LR toxicity on testis. In this paper, we have mainly described the effect of MC-LR; both acute and chronic on the male reproductive system derived from both *in vitro* and *in vivo* studies (Fig. 3). MC-LR was observed to mediate its toxic effect on testis mainly by oxidative stress and DNA damage induced apoptosis in spermatogonia, Leydig cells, Sertoli cells and also *via* affecting the motility and morphology of sperm, and altering hormone regulation of male reproductive system of mice. Thus, MC-LR has the potential to induce toxicity of the male reproductive system.

## Conflict of interest

The authors declare no conflict of interest with respect to this article.

## Transparency document

Transparency document
